# Quantitation of substitutions at amino acid 70 in hepatitis C virus genotype 1b

**DOI:** 10.1186/1743-422X-11-148

**Published:** 2014-08-15

**Authors:** Zhongjie Hu, Ying Liu, Lixia Qiu, Zuopeng Fan, Wei Nie, Shan Liang, Ronghua Jin

**Affiliations:** Department of Hepatitis C & Toxic liver diseases, Beijing Youan Hospital, Capital Medical University, No. 8 Xitoutiao, Youanmenwai, Fengtai District, Beijing, 100069 the People’s Republic of China

**Keywords:** Hepatitis C virus, Genotype 1b, Amino acid 70 substitutions, Degenerate probes, Inosine, Quantitative PCR

## Abstract

**Background:**

Substitutions of amino acid (aa) 70 in the core region of hepatitis C virus genotype 1b (HCV 1b) are a predictor of the non-virological response to pegylated interferon plus ribavirin (PEG-IFN/RBV) therapy. The aim of our study was to develop quantitative real-time reverse transcription polymerase chain reaction (qPCR) assays to quantify wild-type (70 W) and mutant (70 M) strains of HCV 1b.

**Methods:**

We used the TaqMan system to quantify strains 70 W and 70 M. Codon 70 in the HCV 1b core region can be either CGN or CAN, therefore degenerate TaqMan minor groove binder (MGB) probes with inosine were used. We determined detection limits, sensitivity and specificity of the methods developed. Direct sequencing and cloning of the HCV core region were used to confirm the reliability of our new system. Serum samples from 138 Chinese patients infected with HCV 1b were examined with the system we developed and compared with results obtained from the Roche TaqMan RT-PCR HCV RNA quantitation system.

**Results:**

Degenerate MGB probes were able to clearly distinguish 70 W from 70 M. The detection limit was 10^3^ copies/mL. Cross-reactivity tests confirmed the specificity of our method. Our system can effectively quantify 70 W and 70 M for 99.6% of patients with HCV 1b. Further tests involving cloning and sequencing confirmed the reliability of our system.

**Conclusions:**

We developed an assay system using degenerate TaqMan MGB probes with inosine to quantify wild-type and mutant viral RNAs of the HCV 1b core region at aa 70. Our developed assay system had high levels of sensitivity and accuracy, and could prove useful in investigating dynamic changes during PEG-IFN/RBV therapy to assess virological responses.

## Background

Hepatitis C virus (HCV) infection represents a major public health problem as it is a major cause of chronic hepatitis. Considerable numbers of HCV patients develop liver cirrosis and hepatocellular carcinoma (HCC). Over the last 20 years, several antiviral drugs have been developed to treat chronic hepatitis C, including pegylated interferon (PEG-IFNα), ribavirin (RBV) and direct-acting antivirals (DAAs). Until 2011, the combination of PEG-IFNα and RBV (PEG-IFNα/RBV) was the approved treatment for chronic hepatitis C [1]. New therapeutic strategies with DAAs aim towards higher efficacy, pan-genotypic activity, shortened treatment duration, easier administration and improved tolerability and patient adherence [2].

IFN-sparing and -free regimens with or without ribavirin will enter clinical practice in the next few years. However, in some developing countries, such as China, or in some rural and poor regions, use of PEG-IFNα/RBV still represents a standard treatment approach and is sometimes the only available therapy. The long-term response to this therapy remains unsatisfactory, especially in patients infected with HCV genotype 1b (HCV 1b). Additionally, PEG-IFN/RBV combination therapy is accompanied by adverse events. Prediction of the response to antiviral treatment before initiation or in the early phase of therapy would be advantageous. Several factors have been associated with responses to PEG-IFN/RBV therapy; some are associated with the host, such as interleukin 28B single nucleotide polymorphisms (IL28B SNPs), gender, race, age, and obesity [3–7], while some are virus-associated, such as viral genotype, viral load, amino acid (aa) substitution in the core and non-structural (NS) 5A region [8–10].

Substitution at aa 70 of the core protein is one of the most important factors, along with IL28B polymorphisms [11–15]. For patients with the IL28B rs8099917 genotype, 12% of those with Gln70 (His70) had a sustained viral response (SVR), while 50% of patients with Arg70 were conferred an SVR [16]. Detection of aa 70 substitutions is important to predict an early non-virological response (NVR) and is also the basis to study the mechanisms of IFN treatment resistance. To investigate wild-type (Arg70; 70 W) and mutant (Gln/His70; 70 M) strains of HCV 1b, several methods are available including direct sequencing, mutation-specific polymerase chain reaction (PCR) assays [17] and quantitative PCR assays [18]. However, the sensitivity and specificity of these tests are limited. These methods are insufficient for quantifying viral RNAs and cannot be used to investigate the dynamic response of 70 W and 70 M to antiviral therapy.

We attempted to develop an absolute quantitation system for 70 W and 70 M using degenerate TaqMan minor groove binder (MGB) probes with inosine. We also sought to determine the sensitivity, specificity, and reproducibility of our improved system and correlate our results with those from direct sequencing and cloning sequencing.

## Results

### Specificity of probes and optimization of qPCR assays

**Figure 1:**
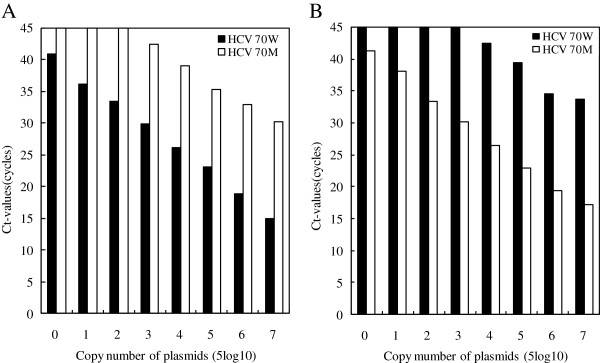
**Quantitation of serial 10-fold dilutions of 70 W and 70 M plasmids using wild-type and mutant probes. A**. Wild-type probe. **B**. Mutant probe.

Probes could detect plasmid templates with one-base mismatch; however, reaction efficiency was lower than that when well-matched plasmids were used. When 5 × 10^7^ copies of the wild-type plasmids (CGG of codon 70) were tested using the mutant probe (CAI), which had a base mismatch, approximately 30 cycles were required before the crossing threshold was reached (Figure 1A). This compares with 15 cycles for the matching probe (CGI). When fewer than 5 × 10^2^ copies of the wild-type plasmid were tested using the mutant probe, no signal was detected by 45 cycles. All serial dilutions of the templates could be detected by the matched probe by 41 cycles. When 5 × 10^7^ copies of the mutant plasmid (CAG of codon 70) template were used in conjunction with the wild-type probe (CGI), approximately 34 cycles were required before the crossing threshold was reached, while only 17 cycles were required for the matching probe (CAI). When fewer than 5 × 10^3^ copies of the mutant plasmid were tested with the wild-type probe, no signal was detected by cycle 45. All serially diluted templates could be detected by the matching probe in 42 cycles (Figure 1B). The slope of the curve for Rn *vs.* cycles for the mismatched reaction was lower than that of the well-matched reaction.

To eliminate the impact of non-specific amplifications, when qPCR assays were prepared, a cross-reaction control with 5 × 10^7^ copies of a one-base mismatch plasmid was used. When data were analyzed, a manual baseline was used to cover the curve vertex of the cross-reaction control to ensure specificity of the system.

### Sensitivity and detection limits of the probes

**Figure 2:**
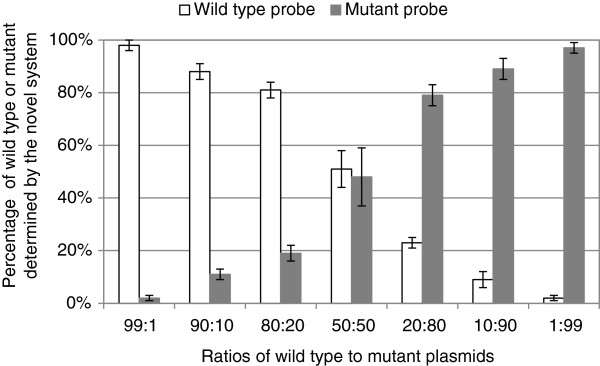
**Relative ratios of wild-type to mutant as determined by the novel detection system.**

The wild-type and mutant plasmids were mixed at ratios of 99:1, 90:10, 80:20, 50:50, 20:80, 10:90, and 1:99, corresponding to final concentrations of 10^7^–10^1^ copies/μL. Using the plasmid mixture as a template, qPCRs were performed. In all cases, wild-type probes could successfully detect 1% of wild-type plasmids in the 100 copies/μL mixture, or 10% for the 10 copies/μL mixture (Table 1). Similarly, 1% of the mutants among the 100 copies/μL mix, or 10% of the 10 copies/μL mixture, were successfully detected with the mutant probes. The calculated relative ratios agreed with those of the template plasmids (Figure 2).

**Table 1:** **Detection limits when using the plasmid mixture as templates**

Probes	W:M plasmid ratio	Plasmid concentration (copies/μL)						
		10 ^1^	10 ^2^	10 ^3^	10 ^4^	10 ^5^	10 ^6^	10 ^7^
Wild-type	99:1	D	D	D	D	D	D	D
	90:10	D	D	D	D	D	D	D
	80:20	D	D	D	D	D	D	D
	50:50	D	D	D	D	D	D	D
	20:80	D	D	D	D	D	D	D
	10:90	D	D	D	D	D	D	D
	1:99	U	D	D	D	D	D	D
Mutant	99:1	U	D	D	D	D	D	D
	90:10	D	D	D	D	D	D	D
	80:20	D	D	D	D	D	D	D
	50:50	D	D	D	D	D	D	D
	20:80	D	D	D	D	D	D	D
	10:90	D	D	D	D	D	D	D
	1:99	D	D	D	D	D	D	D

W:M, ratio of wild-type to mutant plasmids.

D, detectable.

U, undetectable.

### Quantitation of aa 70 substitutions in clinical samples

HCV 70 W and 70 M in serum samples from 138 Chinese patients with HCV 1b were successfully quantified. Sequences from the HCV core region were confirmed by direct sequencing. Using relative ratios, we identified three categories to compare by direct sequencing: less than 1% (wild-type); 1–99% (mixed); and greater than 99% (mutants; Table 2). We found a mix of genotypes in 94% (129/138) of samples. Approximately 68% (88/129) of cases could be detected only by our novel system. The mutant strain was dominant (>50%) in 23% (32/138) of patients.

**Table 2:** **Prevalence of aa 70 substitutions in 138 Chinese patients with HCV 1b who had not been treated**

Core 70 mutant (%)	Direct sequencing		
	Wild-type	Mixed	Mutant
<1	5	0	0
1-10	43	0	0
10-15	13	1	0
15-20	2	6	0
20-30	1	12	0
30-40	1	14	0
40-50	0	8	0
50-80	0	0	0
80-90	0	0	1
90-99	0	0	27
>99	0	0	4

### Accuracy of our novel system

When the system with normal TaqMan MGB probes was used, 89.1% (123/138) of the sums of wild-type and mutant viral loads were comparable with results from the Roche diagnostic system (Table 3). In 15 cases, the results were less than 20% of those from the Roche system. When our improved system with degenerate probes was used, only a single patient’s result was not comparable with those from the Roche diagnostic system.

**Table 3:** **Ratio of HCV RNA loads as determined by novel systems with degenerate probes or normal MGB probes compared with those determined by Roche Diagnostic Systems**

Ratio of HCV RNA loads	Tested with normal probes (%)	Tested with degenerate probes (%)
<2	94 (68.1)	121 (87.7)
2–5	29 (21.0)	16 (11.6)
>5	15 (10.9)	1 (0.7)

Direct sequencing of the core region showed that 83.3% (115/138) of patients’ core genes completely matched primers used in our novel system. We also found that the core genes in 23 patients contained one or two mismatched sites with the primers. However, only one mismatched site (G162A) was near the 3′ end of the sense primer. For the other 22 cases, the mismatched sites were near the 5′ ends of the sense (A154G, C159T) or antisense (A297G/T, C300T, G302A) primers and exerted very little influence on the PCR results. For 89.9% (124/138) of cases, the major codon 70 types were CGG or CAG; for the other 14 cases, they were CGA, CGT, CAA, CAT, or CAC.

### Accuracy of ratios for wild-type to mutant viral loads

The 14 cases with codon 70 types other than CGG or CAG were selected for TA cloning and sequencing. Our results showed that the ratios of wild-type to mutants as determined by our novel system were comparable with those from the cloning and sequencing experiments. For patients with codon 70 types other than CGG and CAG, systems with degenerate probes containing inosine can fix the errors caused by normal probes (Table 4).

**Table 4:** **Correlation of results from the novel system with degenerate probes, normal probes, the Roche diagnostic system, and from sequencing**

No.	Roche system	Normal probes		Degenerate probes		Cloning sequencing	Matched with normal probes	Matched with degenerate probes
	RNA Load ^$^	Ratio ^*^	Wild-type (%)	Ratio ^*^	Wild-type (%)	Codon 70		
1	7.2	0.19	82.1	1.33	98.5	4CGG/1CAG/14CGA/1 N.D.^#^	4CGG/1CAG	18CGN/1CAN
2	6.3	0.01	0.0	1.27	2.6	13CAA/7CAT	None	20CAN
3	7.0	0.08	4.2	1.38	92.0	2CAG/18CGA	2CAG	2CAN/18CGN
4	6.6	0.07	100.0	0.71	97.5	15CGA/5 N.D.	None	15CGN
5	6.6	0.08	100.0	0.87	100.0	1CGG/19CGT	1CGG	20CGN
6	5.6	0.17	4.8	1.39	2.7	3CAG/17CAA	3CAG	20CAN
7	6.5	0.02	63.2	0.70	92.7	2CAA/16CGA/2 N.D.	None	2CAN/16CGN
8	7.9	0.04	0.0	0.80	0.0	18CAA/2 N.D.	None	18CAN
9	7.1	0.03	2.0	1.48	1.5	19CAC/1 N.D.	None	19CAN
10	6.4	0.13	20.6	1.47	4.2	1CGG/3CAG/16CAA	1CGG/3CAG	1CGN/19CAN
11	5.6	0.12	95.6	1.18	96.4	3CGG/17CGA	3CGG	20CGN
12	7.4	0.08	96.0	1.32	8.0	1CGG/15CAT/4 N.D.	1CGG	1CGN/15CAN
13	6.7	0.08	91.3	0.96	9.8	2CGG/16CAA/2 N.D.	2CGG	2CGN/16CAN
14	6.3	0.18	95.5	1.90	97.5	4CGG/16CGA	4CGG	20CGN

^$^RNA loads (Log_10_ copies/mL) as determined by the Roche Diagnostic System.

^*^Ratio of RNA loads (copies/mL) as determined by our novel system to those determined by the Roche Diagnostic System.

^#^No data available.

## Discussion

The study presented here is based on reports that substitution of arginine by glutamine at aa 70 in the HCV 1b core region is an independent and significant marker associated with an NVR to PEG-IFN/RBV therapy. A significant association between HCV core91 substitutions and treatment outcome has been reported in several studies [8, 19]. However, other studies have described that only a variation at residue 70 is associated with treatment response. The aa 70 substitution has been identified as the most important factor for PEG-IFN/RBV treatments with the exception of IL28B polymorphisms [3, 12, 13, 16].

Direct sequencing is the most accurate method for determining aa 70 substitutions, but sensitivity is limited for samples with mixed genotypes. A minor genotype present at levels of about 15–20% might not be detected by direct sequencing. Direct sequencing is not able to quantify virus RNAs and cannot be used to investigate the dynamic response to antiviral therapy of wild-type and mutant strains. Because the sequencing method is costly, Okamoto et al. [17] developed a new method, the amplification refractory mutation system (ARMS) reverse transcription PCR (RT-PCR), and this became widely adopted. In the HCV 1b core region, several codons encode arginine (CGG/C/U/A) in wild-type proteins; however, the second base is always a “G”. Similarly, the second base of the codon encoding glutamine or histidine (CAG/C/U/A) in mutant proteins is always an “A”. Thus, detection of wild-types or mutants based on a single-base difference can be achieved by designing the common second base (A or G) at the 3′ end of primers. Compared with direct sequencing, the mutation-specific PCR is simpler, quicker, and more convenient with a satisfactory detection rate, frequency of competitive-type cases, and re-examination rate. However, the authors also pointed out that the system excludes the presence of a minor type. Detailed examination, such as sequence analysis using cloning, might be necessary when a competitive type is indicated by the detection system. Moreover, the system is unable to quantify virus RNAs. To quantify the aa 70 mutations, Nakamoto et al. [18] developed a real-time ARMS RT-PCR assay. Mutation-specific primers are able to distinguish the difference between wild-type and mutant plasmids at the same quantitative levels. The real-time ARMS RT-PCR is more convenient because it uses a fluorescent signal instead of restriction enzyme digestion, gel electrophoresis, or sequence analysis of PCR products. Importantly, the system can quantify the proportion of core substitutions. The specificity of the system is dependent upon the mutation-specific primers; a minor genotype might be masked when the ratio is less than 1:100. Furthermore, with the SYBR Green method, primer dimers and non-specific amplification artifacts can decrease specificity.

To overcome these disadvantages, we developed a TaqMan two-step RT-PCR system with high sensitivity and specificity using MGB probes to quantify the main genotypes of aa 70. Our system cannot be used for aa 70 genotypes other than CGG/CAG, which account for about 13% of patients with HCV 1b.

Considering the variability of HCV RNA, primers and probes should be designed to match HCV quasispecies as much as possible. We first determined a consensus sequence of the HCV 1b core gene based on 489 sequences [20]. Primers were then designed based on conserved regions. Our results showed that for 115 of 138 patients, the primers matched well with the HCV core gene. Of the 23 patients with mismatched bases at the primer sites, 22 were near the 5′ end of the primers and had little influence on the PCR results. A single mismatched site (G162A) was near the 3′ end of the sense primer and resulted in reduced PCR efficiency. Because primer mismatches are critical to the success of qPCR assays, primer sequences must be modified if mismatches are frequent. The frequencies of possible primer mismatches, including G162A, were very low, especially for sites near the 3′ end, and possibly lowered PCR efficiency (Table 5). Therefore, the primers designed for the system will work well for the vast majority of patients.

**Table 5:** **Frequencies of possible primer mismatches based on 489 sequences of the HCV 1b core gene**

Sense primer			Anti-sense primer		
nt	5′	Frequencies of mismatch (%)	nt	5′	Frequencies of mismatch (%)
148	A	0.00	302	C	1.64
149	G	0.20	301	G	0.20
150	G	0.41	300	G	3.07
151	A	0.00	299	G	0.00
152	A	0.00	298	G	0.00
153	G	0.20	297	T	7.98
154	A	0.61	296	G	0.00
155	C	0.00	295	A	0.20
156	T	0.00	294	C	0.20
157	T	0.20	293	A	0.20
158	C	0.41	292	G	0.61
159	C	2.04	291	G	0.20
160	G	0.00	290	A	0.00
161	A	0.41	289	G	0.20
162	G	0.41	288	C	0.00
163	C	0.00	287	C	0.00
164	G	0.00	286	A	0.00
165	G	0.20			
166	T	0.61			
167	C	0.41			

For the probes, because the target site was codon 70, the only way to improve specificity is to shorten the length of the probe. The MGB probe was the best option because when it is hybridized to a complementary target, the MGB molecule folds into duplex and hyper-stabilizes, allowing the use of shorter, more specific probe sequences [21]. Although MGB probes can detect single-base mutations, the system we developed can still detect part of plasmid templates with one-base mismatch. The reason for this might be because there were too many G and C bases around codon 70, therefore the G/C to A/T ratio in probes was too high (>80%). Reaction efficiency and crossing threshold were lower than those in corresponding plasmids. The slope of the curve for Rn *vs.* cycles for the mismatched reactions was much lower than those for the well-matched reactions. When the cross-reaction control with 5 × 10^7^ copies of one-base mismatch plasmid was used, a manual baseline could be moved to cover the curve vertex of the cross-reaction control. The curves for the standards were at the exponential growth stage, with parallelism between curves still very good.

When compared with the Roche diagnostic system, which is widely used in clinics, our novel system exhibited good comparative performance. When the system with normal TaqMan MGB probes was used, the results for 15 patients were less than 5-fold from those of the Roche diagnostic system. For one case, it was because there was a mismatched site near the 3′ end of the sense primer. For the other 14 cases, different codon 70 types (CGA, CGT, CAA, CAT, and CAC) were identified by direct sequencing. The normal probes designed for “CGG or CAG” were not strong matches for the sequences. When our improved system with degenerate probes was used, all results were comparable with those from the Roche diagnostic system. The degenerate probes were designed with inosine as the third base of codon 70. Inosine can indiscriminately pair with adenine, thymine, guanine or cytosine. This allows for a probe design that spans SNPs without the polymorphism disrupting the annealing efficiency of probes. To determine the proportion of patients who were suitable for probes, the types of codon 70 for the 489 independent HCV 1b gene sequences were analyzed. Of the codon 70 types, 87.1% are CGG and CAG, and can be effectively tested by the system with normal TaqMan MGB probes. CGN and CAN account for 99.6% of codon 70 types and can be detected with our improved system using degenerate probes (Table 6).

**Table 6:** **Proportions of codon 70 types among 489 independent HCV 1b gene sequences**

Codon 70 types in the HCV 1b core region	Frequency	%
CGG	246	50.3
CAG	180	36.8
CGA	26	5.3
CAA	18	3.7
CAT	12	2.5
CGT	3	0.6
CAC	2	0.4
CCG	1	0.2
CTT	1	0.2
Total	489	100

The detection limit of our assay (1.0 × 10^3^ copies/mL) was not satisfactory. To detect 1% of mutation, samples with viral loads greater than 1.0 × 10^5^ copies/mL would be required. However, it is very difficult to further improve sensitivity in the laboratory. Therefore, the novel detection system is mainly for patients with viral loads greater than 1.0 × 10^5^ IU/mL at baseline. In many published studies, the inclusion criteria involve viral loads greater than 100 kIU/mL [8, 13, 19]. Among the 138 patients tested in the study, only the viral loads of five patients were lower than 1.0 × 10^5^ copies/mL. Our novel assay is useful for the majority of patients. We are now focusing on a larger study, and our novel quantitative detection system is expected to be useful for investigating the dynamic response of wild-types and mutants to PEG-IFNα/RBV therapy, and the effects of R70Q/H substitutions on resistance to PEG-IFNα/RBV therapy.

## Conclusions

We developed a detection system for quantitatively determining aa 70 substitutions in the HCV 1b core region. Our novel developed system had good sensitivity and accuracy, and was helpful in quantifying wild-type and mutant viral RNAs. It also provides a way to investigate the dynamic responses of wild-type and mutant viral RNAs to PEG-IFN/RBV therapy.

## Methods

### Ethics statement

Our study protocol conformed to the ethical guidelines of the Declaration of Helsinki and was approved by The Ethical Committee of Beijing Youan Hospital, Capital Medical University. Written informed consent was obtained from each patient participating in this study.

### Serum samples

Between January 2012 and April 2014, serum samples were obtained from 138 Chinese patients with chronic HCV 1b infection that had not been treated. Patients were from the Department of Hepatology, Beijing Youan Hospital, Capital Medical University and were used to develop and evaluate our novel detection system. Total viral loads were determined by TaqMan qPCR (Amplicor, Roche Diagnostic Systems, Shanghai, China). Samples were kept at −80°C until required.

### HCV RNA extraction and reverse transcription

We extracted HCV RNA from serum samples (140 μL) using a QIAamp Viral RNA Mini Kit according to the manufacturer’s protocol (Qiagen, Shanghai, China). We prepared cDNA by reverse transcription with random hexamers and TaqMan Reverse Transcription Reagents (Applied Biosystems). Reverse transcription reaction conditions involved incubation at 25°C for 10 min, then 42°C for 40 min, and finally 95°C for 5 min.

### Plasmid DNA controls

The HCV core gene was amplified from the serum of a patient with HCV 1b using PCR and specific primers (5′-AAT GCC TGG AGA TTT GGG-3′ and 5′-TTG GAG CAG TCG TTC GTG-3′). Thermal cycling conditions involved denaturation at 95°C for 10 min, followed by 40 cycles of amplification (30 s at 95°C, 30 s at 55°C, and 1 min at 72°C) and a 7-min extension step at 72°C after the 40^th^ cycle. Amplicons were purified using a QIAquick PCR Purification Kit (Qiagen) after agarose gel electrophoresis, and then cloned into pCR-TOPO2.1 (Invitrogen) according to the manufacturer’s protocol. Positive clones were picked and codon 70 types were identified by sequencing (Beijing AuGCT DNA-SYN Biotechnology Co., Ltd). The codon 70 wild-type was CGG. We then made a plasmid carrying a mutant codon 70 (CAG). Site-directed mutagenesis was performed using a QuikChange II Site-Directed Mutagenesis Kit (Agilent Technologies, Santa Clara, CA, USA) according to the manufacturer’s protocol and specific complementary primers (5′-CAA GGC TCG CCG GCC CGA GGG CAG GGC CTG-3′ and 5′-CAG GCC CTG CCC TCG GGC CGG CGA GCC TTG-3′). The introduction of the mutation was confirmed by sequencing. Plasmids containing wild-type or mutant codon 70 were serially diluted 10-fold to provide a dilution range of 10^7^–10° copies/μL.

### HCV 1b core region consensus sequence and codon 70 types

We downloaded 1336 HCV 1b gene sequences, including the full-length core region, from the European HCV database (euHCVdb), HCV Databases from Los Alamos National Laboratory, HCV Database (HCVdb) from the Viral Bioinformatics Resource Center (VBRC), and National Center for Biotechnology Information (NCBI) [20]. Of these, 847 sequences were excluded (814 were from repeated cases at different time points, 19 from cultured cells, eight from plasmids, and six from animal experiments). The remaining 489 sequences were used to determine the consensus sequence of the HCV 1b core region and codon 70 types with the GENETYX software application (Genetyx Corp., Tokyo, Japan).

### Primer and TaqMan probe design for qPCR assays

Primers were synthesized by Sangon Biotech (Shanghai) Co. Ltd. Compared with the TaqMan MGB probes for the main types of codon 70 (CGG and CAG), inosine was used to design two degenerate TaqMan MGB probes for 70 W (CGN) and 70 M (CAN) (Table 7). Probes were purchased from Invitrogen (Shanghai). We performed qPCR assays in a final volume of 50 μL; each reaction contained 5 μL of cDNA, 0.3 μM each primer, 0.1 μM probe and 25 μL of 2× LightCycler® 480 Probes Master Mix (Roche Applied Science, Shanghai, China). Two separate reactions were prepared to detect the wild-types and mutants, but were simultaneously carried out on the same thermal cycling system. Thermal cycling conditions involved an initial denaturation step at 95°C for 10 min, followed by 45 cycles of amplification (15 s at 95°C and 1 min at 60°C). All assays were performed in triplicate on a LightCycler® 480 real-time PCR System (Roche Applied Science) and results were analyzed using LightCycler® 480 Software (Roche Applied Science).

**Table 7:** **Primers and TaqMan MGB probes used for detection of aa 70 substitutions**

Type	Primer name	Binding position (nt)	Sequence (5′–3′)
Primers	sense	148–167	AGG AAG ACT TCC GAG CGG TC
	antisense	302–286	CGG GGT GAC AGG AGC CA
Degenerate probes	70 W(CGN)	203–217	FAM-CTC GCC GIC CCG AGG-MGB
	70 M(CAN)	203–219	VIC-CTC GCC AIC CCG AGG GC-MGB
Normal probes	70 W(CGG)	204–217	FAM-TCG CCG GCC CGA GG-MGB
	70 M(CAG)	203–218	VIC-CTC GCC AGC CCG AGG G-MGB

CR, core region; nt, nucleotides; I, inosine.

### Nucleotide sequencing of the HCV core gene

We used direct sequencing to determine the HCV core gene sequence from the sera of 138 patients. Nucleic acids were amplified by PCR using specific primers (5′-AAT GCC TGG AGA TTT GGG-3′ and 5′-TTG GAG CAG TCG TTC GTG-3′). All samples were initially denatured at 95°C for 10 min and then subjected to 40 cycles of amplification (30 s at 95°C, 30 s at 55°C, and 1 min at 72°C), with an additional 7 min final extension step at 72°C. Amplicons were purified with a QIAquick PCR Purification Kit (Qiagen) after agarose gel electrophoresis and then used for direct sequencing (Beijing AuGCT DNA-SYN Biotechnology Co. Ltd).

The ratio of 70 W to 70 M was determined by cloning and sequencing for some of the patients and results compared with those for the improved system to evaluate accuracy. Cloning was carried out using TOPO TA Cloning Kits (Invitrogen) according to the manufacturer’s protocol. We conducted PCRs using 5′-TCG TGG AAG GCG ACA ACC-3′ and 5′-GCC GAC GAG CGG AAT GT-3′ as sense and antisense primers, respectively. We then randomly picked 20 colonies for each sample and had them sequenced (Beijing AuGCT DNA-SYN Biotechnology Co. Ltd).
